# Supplementary material for: cAMP and c-di-GMP synergistically support biofilm maintenance through the direct interaction of their effectors
Source: Nat Commun. 2022 Mar 21;13:1493. doi: 10.1038/s41467-022-29240-5 (PMC8938473; doi:10.1038/s41467-022-29240-5)
Supplement: Supplementary file 1 — Supplementary Information [file 41467_2022_29240_MOESM1_ESM.pdf]

Supplementary Information

cAMP and c-di-GMP synergistically support biofilm maintenance through the direct interaction of their effectors

Cong Liu[1,2], Di Sun[1,2], Jiawen Liu[1], Ying Chen[1], Xuge Zhou[1], Yunrui Ru[1], Jingrong Zhu[1], Weijie Liu[1,*]

[1]Jiangsu Key Laboratory of Phylogenomics & Comparative Genomics, School of Life Science, Jiangsu Normal University, Xuzhou, China

[2]These authors contributed equally: Cong Liu, Di Sun.

*Correspondence:

Weijie Liu, Tel: +86 18252161264; E-mail: leonliu2013@126.com (WJ Liu)

Postal address of corresponding author, Weijie Liu

No.101, Shanghai road, Tongshan new district, Xuzhou 221116, Jiangsu Province, China

Supplementary Figures

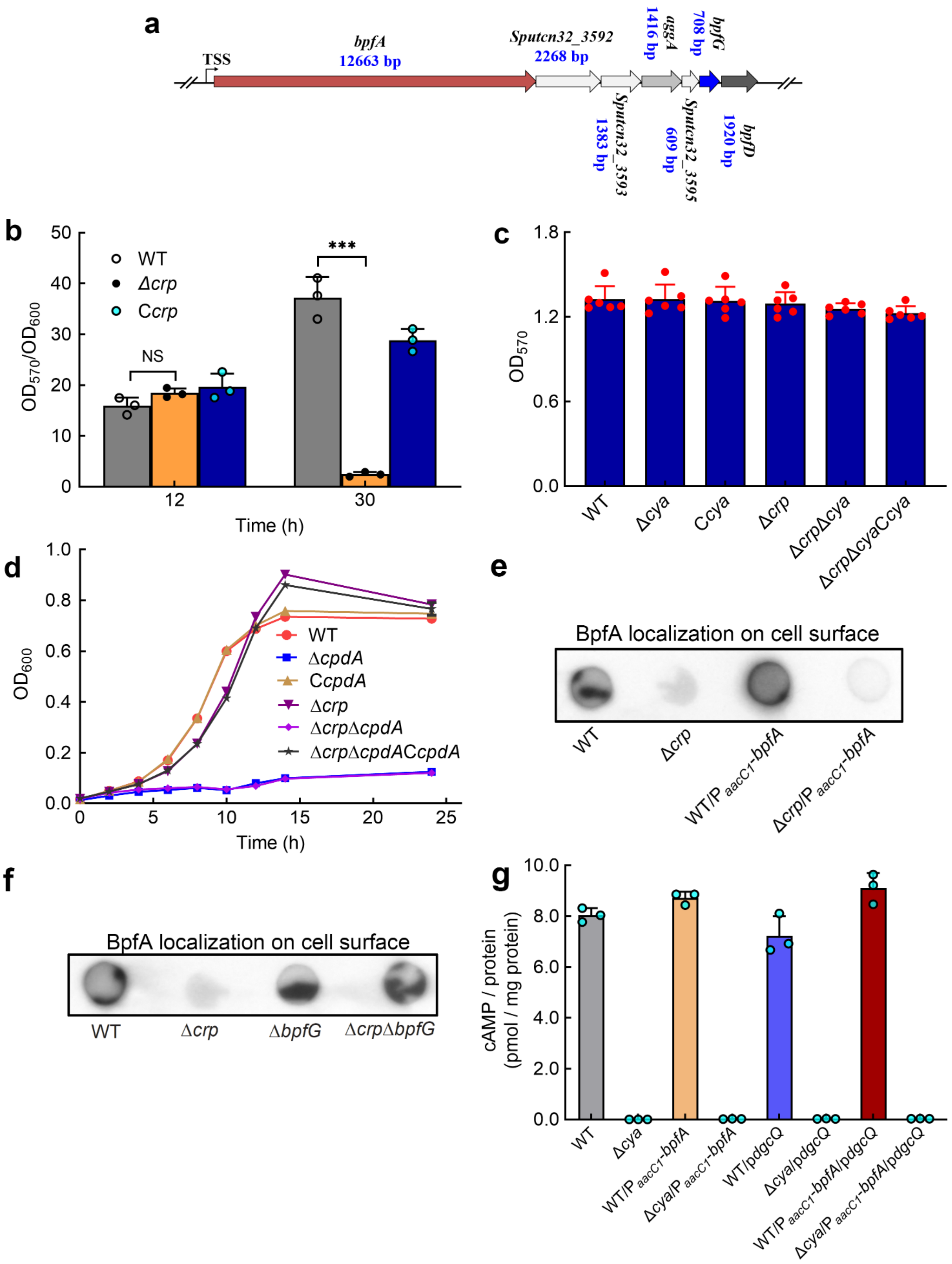

Supplementary Fig. 1 **a** Schematic diagram of the *bpfA* operon. **b** The ratio of biofilm biomass to cell growth (OD_{570}/OD_{600}) at 12 h and 30 h (n=3 independent samples). **c** Biofilm biomass at 12 h

(n=6 independent samples). **d** Cell growth of *cpdA*-related strains (n=3 independent samples). **e, f** BpfA localization on the cell surface detected by dot blotting analysis. **g** Intracellular cAMP concentration (n=3 independent samples). Data in **b, c, d, g** are shown as the mean ± SD. Two-sided Student's *t*-test was used in **b** to analyze the statistical significance (NS: No significance. ***: $p < 0.001$). Source data are provided as a Source Data file.

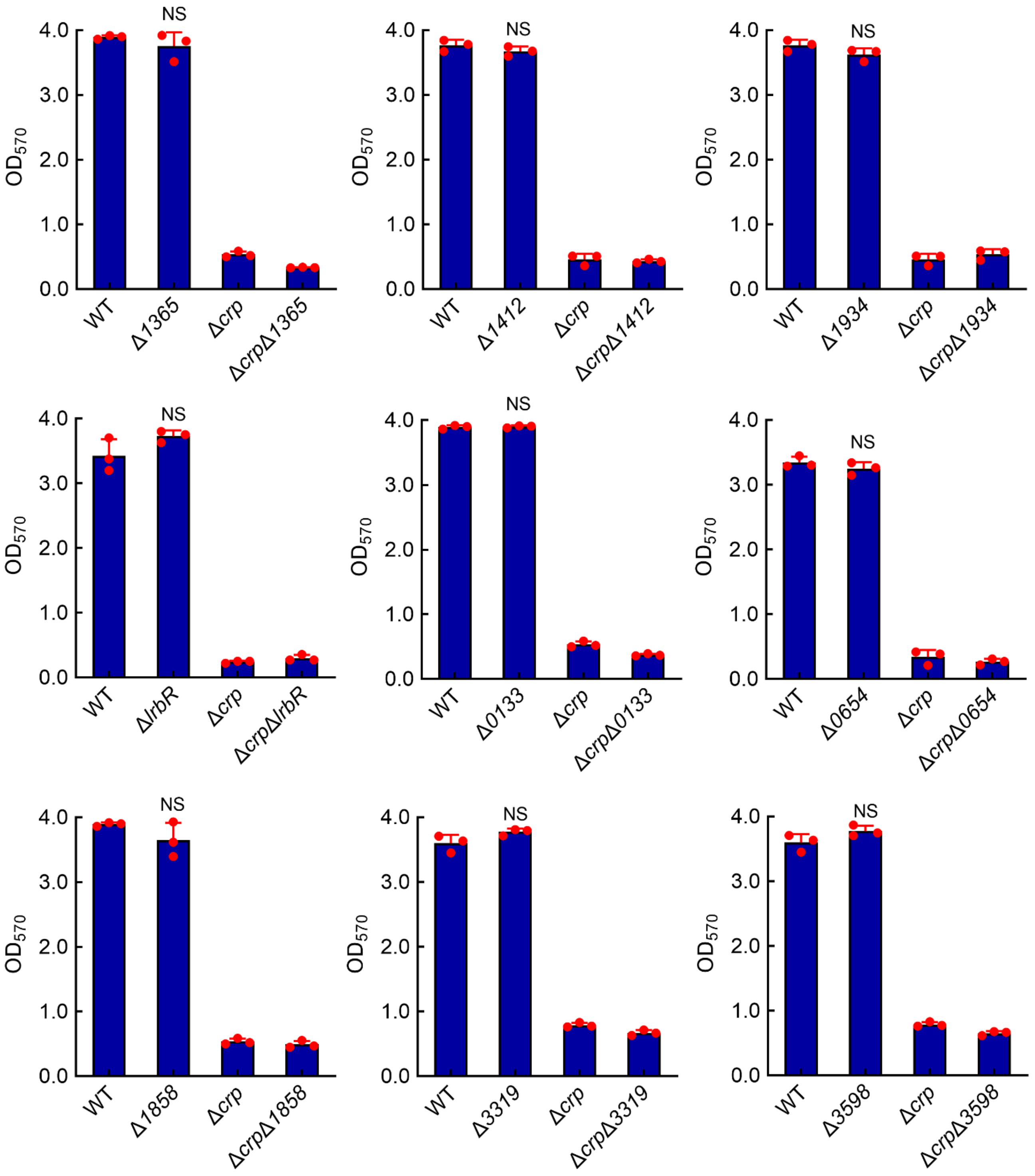

Supplementary Fig. 2 The biofilm biomasses at 30 h of mutants with deletion of *dgc/pde* genes whose transcription level in Δ*crp* changed more than 2.5-fold compared to WT. Data are shown as the mean ± SD (n=3 independent samples). Two-sided Student's *t*-test was used to analyze the statistical significance (NS: No significance). Source data are provided as a Source Data file.

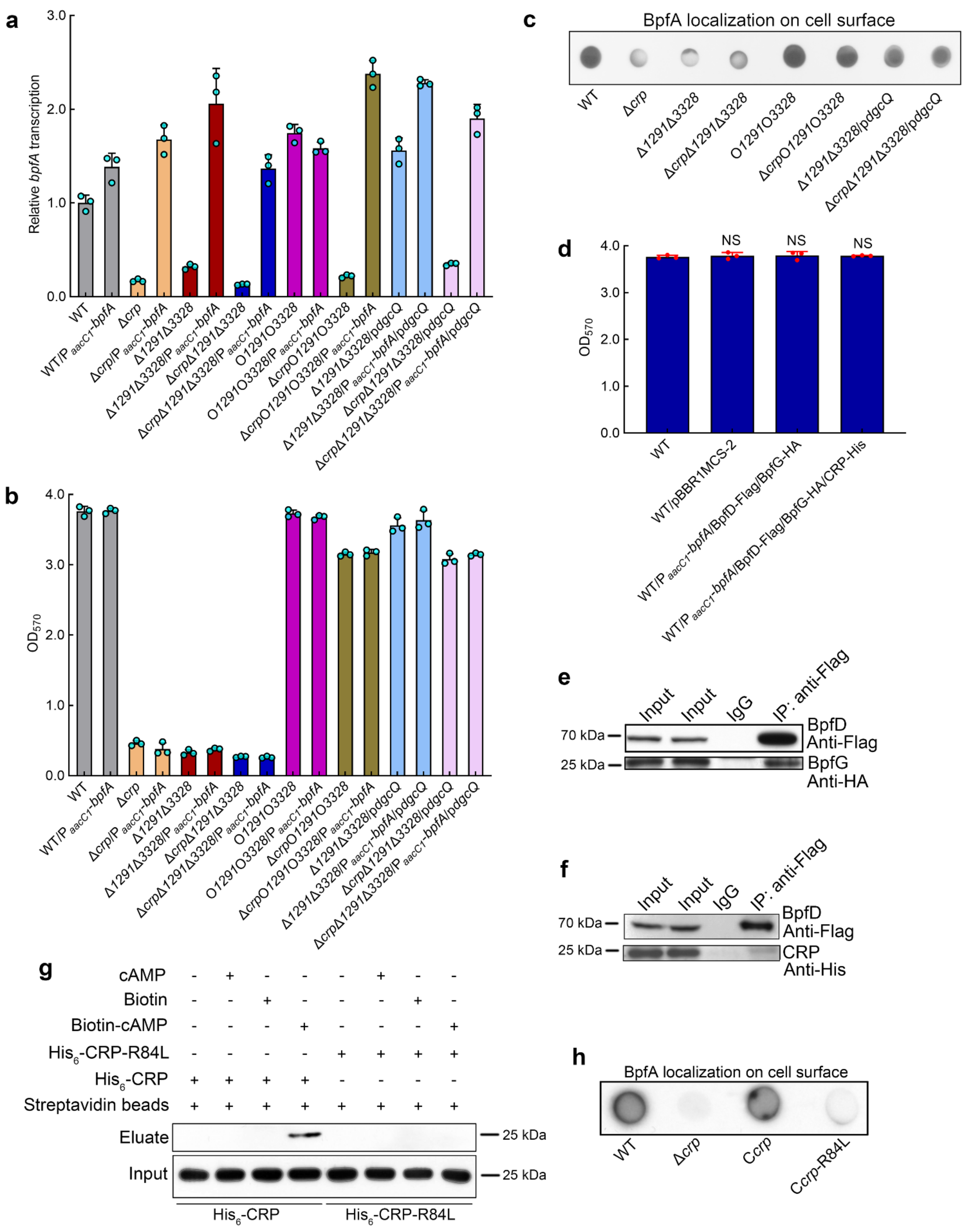

Supplementary Fig. 3 **a** Comparison of *bpfA* transcription. **b** Biofilm biomass. **c** BpfA localization on the cell surface detected by dot blotting analysis. **d** Biofilm biomass. **e, f** Co-IP using antibodies against BpfD, BpfG, CRP, and immunoglobulin G (IgG) negative control. **g** Interaction between cAMP and CRP and CRP-R84L by biotinylated cAMP pull-down assay. **h** BpfA localization on the cell surface detected by dot blotting analysis. Data in **a, b, d** are shown as the mean ± SD (n=3

independent samples). Two-sided Student's *t*-test was used in **d** to analyze the statistical significance (NS: No significance). Source data are provided as a Source Data file.

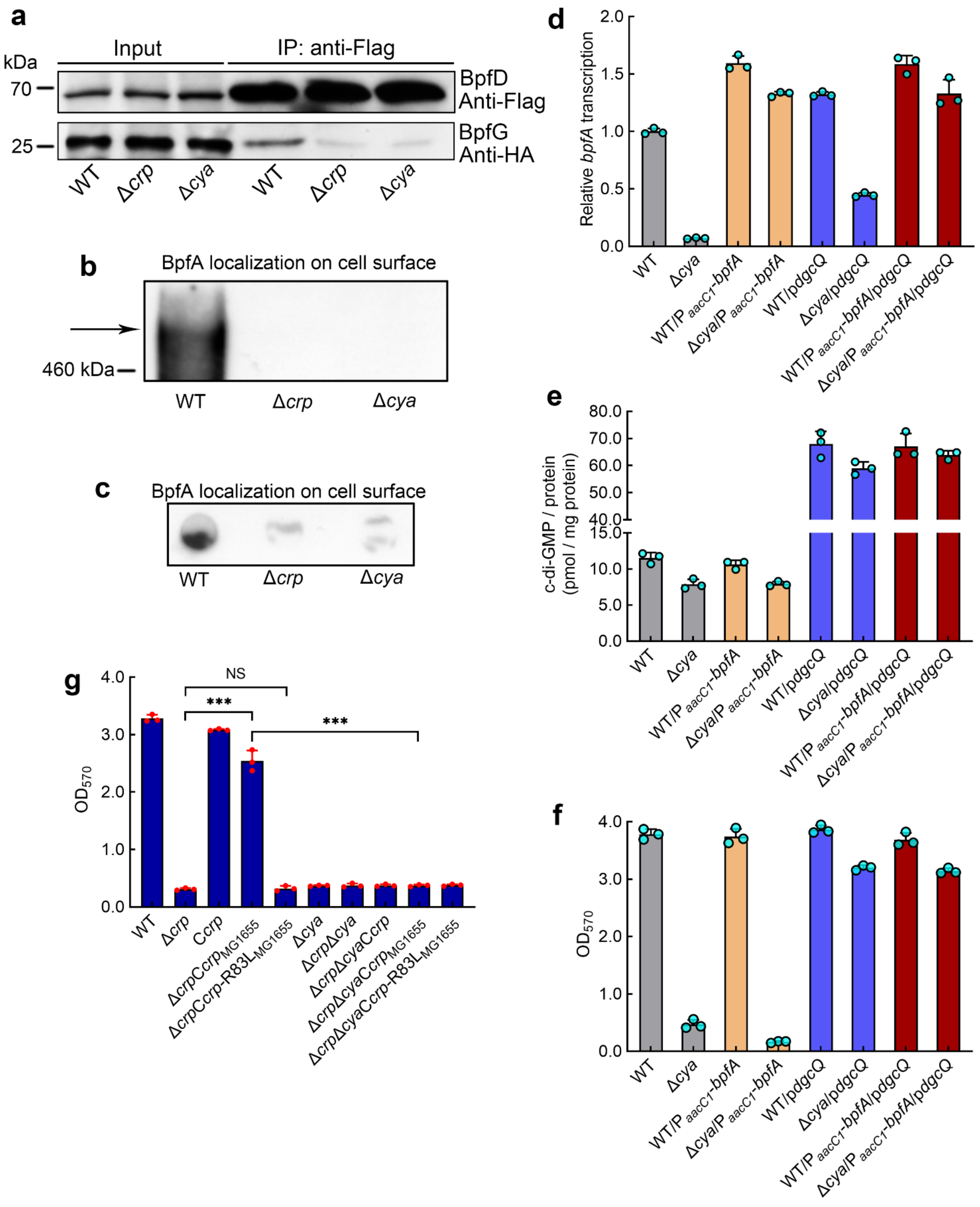

Supplementary Fig. 4 a The interaction between BpfD and BpfG by Co-IP. **b** The BpfA localization on the cell surface detected by western blotting. **c** The BpfA localization on the cell surface detected by dot blotting. The native promoter of the *bpfA* operon in all strains (**a-c**) was replaced by the constitutive promoter P_{aacC1}. All strains (**a-c**) were cultured for 30 h in the biofilm state. **d** Transcriptional analysis of *bpfA*. **e** Intracellular c-di-GMP concentration. **f, g** Biofilm biomass. Data

in **d, e, f, g** are shown as the mean ± SD (n=3 independent samples). Two-sided Student's *t*-test in **g** was used to analyze the statistical significance (***: $p < 0.001$, NS: No significance). Source data are provided as a Source Data file.

Supplementary Tables

Supplementary Table 1 Transcriptional analysis of *dgc* and *pde* genes in WT and Δ*crp* at 30 h.

Gene and enzyme category	Fold change	*p* value
Protein containing a GGDEF domain		
*Sputcn32*_0384	1.981	1.04E-04
*Sputcn32*_0414	1.846	1.81E-04
*Sputcn32*_0498	2.195	1.41E-04
*Sputcn32*_1039	1.668	2.44E-03
*Sputcn32*_1235	1.886	6.16E-05
*Sputcn32*_1365[b]	2.676	2.64E-04
*Sputcn32*_1412[b]	13.332	3.72E-06
*Sputcn32*_1741	1.152	3.72E-02
*Sputcn32*_1934[b]	3.011	1.33E-03
*Sputcn32*_2096	1.974	1.07E-03
*Sputcn32*_2671	0.978	9.03E-01
*Sputcn32*_3018	1.922	6.02E-05
*Sputcn32*_3085	1.598	4.07E-04
*Sputcn32*_3168	1.443	4.81E-02
*Sputcn32*_3244	1.827	5.45E-06
*Sputcn32*_3269	1.201	7.45E-02
*Sputcn32*_3306	1.222	2.33E-02
*Sputcn32*_3390	2.107	6.46E-06
Protein containing an EAL domain		
lrbR[b]	7.611	3.45E-07
*Sputcn32*_0601	1.309	1.68E-02
*Sputcn32*_0814	1.651	6.08E-04
*Sputcn32*_1344	2.351	7.85E-05
*Sputcn32*_1988	2.172	4.27E-03
*Sputcn32*_2106	1.376	2.26E-03
*Sputcn32*_2362	1.663	7.45E-05
*Sputcn32*_3141	1.500	1.36E-05
Protein containing a dual GGDEF/EAL domain		
*Sputcn32*_0099	1.421	3.50E-05
*Sputcn32*_0133[b]	2.588	3.39E-04
*Sputcn32*_0327	2.023	7.81E-04
*Sputcn32*_0555	1.868	5.47E-04
*Sputcn32*_0654[b]	2.529	1.74E-04
*Sputcn32*_1253	1.034	5.46E-01
*Sputcn32*_1800	2.016	1.35E-03
*Sputcn32*_1851	1.276	7.53E-04
*Sputcn32*_1858[b]	2.746	2.78E-03
*Sputcn32*_1917	0.969	4.17E-01
*Sputcn32*_2456	1.146	7.17E-02
*Sputcn32*_2800	0.890	2.06E-03
*Sputcn32*_2830	1.202	4.29E-04
*Sputcn32*_3319[b]	4.319	4.83E-05

*Sputcn32*_3405	1.071	1.83E-01
*Sputcn32*_3598[b]	8.402	8.89E-05
*Sputcn32*_3648	0.806	4.97E-03
*Sputcn32*_3856	1.345	7.03E-03
*Sputcn32*_3917	1.202	1.14E-03

[a] Fold change data indicate the WT/Δ*crp* ratios for genes responsible for the expression of proteins involved in the regulation of c-di-GMP. [b] The WT/Δ*crp* ratio more than 2.5 folds. The *p* value indicates the level of statistical significance of the differential expression. The statistical significance was determined by two-sided Student's *t*-test.

Supplementary Table 2 Strains and plasmids used in this study.

Strains and mutants	Usages or Descriptions	Sources
Escherichia coli		
DH5α	Routine cloning host. *fhuA2*, *lacΔU169*, *phoA*, *glnV44*, *Φ80'*, *lacZΔM15*, *gyrA96*, *recA1*, *relA1*, *endA1*, *thi-1*, *hsdR17*	Lab stock
BL21(DE3)	Protein expression host. *dcm*, *ompT*, *hsdS* ($r_B^- m_B^-$) *gal*	Lab stock
UQ3021	DH5α/*λpir*	Lab stock
UQ3022	Donor strain for the mini-Tn5 transposon containing plasmid, pRL27	
BL21(DE3)/pET28a-CRP	Heterologous expression of N-terminal His_6-tagged *S. putrefaciens* CN32 CRP protein	This study
BL21(DE3)/pET28a-CRP-R84L	Heterologous expression of N-terminal His_6-tagged *S. putrefaciens* CN32 site-directed CRP-R84L protein	This study
BL21(DE3)/pGEX-4T-1-BpfD-In	Heterologous expression of N-terminal GST-tagged BpfD of intracellular domain	This study
BL21(DE3)/pGEX-4T-1	Expression of GST protein	This study
BL21(DE3)/pMAL-c2x-1291	Heterologous expression of N-terminal MBP-tagged Sputcn32_1291 protein	This study
BL21(DE3)/pMAL-c2x-1291-GGAAF	Heterologous expression of N-terminal MBP-tagged site-directed Sputcn32_1291GGAAF protein	This study
Shewanella putrefaciens CN32		
Wild type (WT)	*Shewanella putrefaciens* CN32	51
WT/pBBR1MCS-2	CN32 carrying plasmid pBBR1MCS-2	This study
Δ*crp*	CN32 Δ*crp* (*Sputcn32_0652*)	This study
C*crp*	CN32Δ*crp* carrying complement pBBR1MCS-2-P_{aacC1}-*crp*; Km^r	This study
Δ*crp*C*crp*$_{MG1655}$	CN32Δ*crp* carrying complement pBBR1MCS-2- P_{aacC1}-*crp*$_{MG1655}$; Km^r	This study
Δ*crp*C*crp*-R83L$_{MG1655}$	CN32Δ*crp* carrying complement pBBR1MCS-2- P_{aacC1}-*crp*-R83L$_{MG1655}$; Km^r	This study
Δ*cya* (Δ*cyaA*Δ*cyaB*Δ*cyaC*)	CN32 Δ*cyaA*(*Sputcn32_3586*) Δ*cyaB*(*Sputcn32_3104*) Δ*cyaC*(*Sputcn32_1140*)	This study
C*cya* (C*cyaA*-C*cyaB*-C*cyaC*)	Δ*cya* carrying complement pBBR1MCS-2-P_{aacC1}-*cyaA*-*cyaB*-*cyaC*; Km^r	This study
Δ*crp*Δ*cya*	CN32 Δ*crp*Δ*cyaA*Δ*cyaB*Δ*cyaC*	This study
Δ*crp*Δ*cya*C*cya*	CN32 Δ*crp*Δ*cyaA*Δ*cyaB*Δ*cyaC* carrying complement pBBR1MCS-2-P_{aacC1}-*cyaA*-*cyaB*-*cyaC*; Km^r	This study
Δ*crp*Δ*cya*C*crp*	CN32 Δ*crp*Δ*cyaA*Δ*cyaB*Δ*cyaC* carrying complement pBBR1MCS-2-P_{aacC1}-*crp*; Km^r	This study
Δ*crp*Δ*cya*C*crp*$_{MG1655}$	CN32 Δ*crp*Δ*cyaA*Δ*cyaB*Δ*cyaC* carrying complement pBBR1MCS-2-P_{aacC1}-*crp*$_{MG1655}$; Km^r	This study
Δ*crp*Δ*cya* C*crp*-R83L$_{MG1655}$	CN32 Δ*crp*Δ*cyaA*Δ*cyaB*Δ*cyaC* carrying complement pBBR1MCS-2-P_{aacC1}-*crp*-R83L$_{MG1655}$; Km^r	This study
Δ*cpdA*	CN32 Δ*cpdA*(*Sputcn32_0771*)	This study
C*cpdA*	CN32 Δ*cpdA* carrying complement pBBR1MCS-2-P_{aacC1}-*cpdA*; Km^r	This study
Δ*crp*Δ*cpdA*	CN32Δ*crp*Δ*cpdA*	This study
Δ*crp*Δ*cpdA*C*cpdA*	CN32 Δ*crp*Δ*cpdA* carrying complement pBBR1MCS-2-P_{aacC1}-*cpdA*; Km^r	This study
Δ*bpfA*	CN32 Δ*bpfA*(*Sputcn32_3591*)	This study
Δ*crp*Δ*bpfA*	CN32Δ*crp*Δ*bpfA*	This study
Δ*cya*Δ*bpfA*	CN32Δ*cyaA*Δ*cyaB*Δ*cyaC*Δ*bpfA*	This study

Δ*crp*Δ*cya*Δ*bpfA*	CN32Δ*crp*Δ*cyaA*Δ*cyaB*Δ*cyaC*Δ*bpfA*	This study
Δ*bpfD*	CN32 Δ*bpfD*(*Sputcn32_3597*)	This study
Δ*crp*Δ*bpfD*	CN32 Δ*crp*Δ*bpfD*	This study
Δ*bpfG*	CN32 Δ*bpfG*(*Sputcn32_3596*)	This study
Δ*crp*Δ*bpfG*	CN32 Δ*crp*Δ*bpfG*	This study
WT/p*dgcQ*	CN32 carrying pBBR1MCS-2-P_{aacC1}-*dgcQ*$_{MG1655}$; Km^r	This study
Δ*crp*/p*dgcQ*	CN32 Δ*crp* carrying pBBR1MCS-2-P_{aacC1}-*dgcQ*$_{MG1655}$; Km^r	This study
Δ*cya*/p*dgcQ*	CN32 Δ*cyaA*Δ*cyaB*Δ*cyaC* carrying pBBR1MCS-2-P_{aacC1}-*dgcQ*$_{MG1655}$; Km^r	This study
Δ*0133*	CN32 Δ*Sputcn32_0133*	This study
Δ*crp*Δ*0133*	CN32 Δ*crp*Δ*Sputcn32_0133*	This study
Δ*lrbR*	CN32 Δ*lrbR*(*Sputcn32_0305*)	51
Δ*crp*Δ*lrbR*	CN32 Δ*crp*Δ*lrbR*	This study
Δ*0654*	CN32 Δ*Sputcn32_0654*	This study
Δ*crp*Δ*0654*	CN32 Δ*crp*Δ*Sputcn32_0654*	This study
Δ*1291*	CN32 Δ*Sputcn32_1291*	This study
Δ*crp*Δ*1291*	CN32 Δ*crp*Δ*Sputcn32_1291*	This study
Δ*1365*	CN32 Δ*Sputcn32_1365*	This study
Δ*crp*Δ*1365*	CN32 Δ*crp*Δ*Sputcn32_1365*	This study
Δ*1412*	CN32 Δ*Sputcn32_1412*	This study
Δ*crp*Δ*1412*	CN32 Δ*crp*Δ*Sputcn32_1412*	This study
Δ*1858*	CN32 Δ*Sputcn32_1858*	This study
Δ*crp*Δ*1858*	CN32 Δ*crp*Δ*Sputcn32_1858*	This study
Δ*1934*	CN32 Δ*Sputcn32_1934*	This study
Δ*crp*Δ*1934*	CN32 Δ*crp*Δ*Sputcn32_1934*	This study
Δ*3319*	CN32 Δ*Sputcn32_3319*	This study
Δ*crp*Δ*3319*	CN32 Δ*crp*Δ*Sputcn32_3319*	This study
Δ*3328*	CN32 Δ*Sputcn32_3328*	This study
Δ*crp*Δ*3328*	CN32 Δ*crp*Δ*Sputcn32_3328*	This study
Δ*3598*	CN32 Δ*Sputcn32_3598*	This study
Δ*crp*Δ*3598*	CN32 Δ*crp*Δ*Sputcn32_3598*	This study
Δ*1291*Δ*3328*	CN32 Δ*Sputcn32_1291*Δ*Sputcn32_3328*	This study
Δ*1291*Δ*3328*C*1291*C*3328*	CN32 Δ*Sputcn32_1291*Δ*Sputcn32_3328* carrying complement pBBR1MCS-2-P_{aacC1}-*Sputcn32_1291*- *Sputcn32_3328*; Km^r	This study
Δ*crp*Δ*1291*Δ*3328*	CN32 Δ*crp*Δ*Sputcn32_1291*Δ*Sputcn32_3328*	This study
Δ*crp*Δ*1291*Δ*3328*C*1291*C*3328*	CN32 Δ*crp*Δ*Sputcn32_1291*Δ*Sputcn32_3328* carrying complement pBBR1MCS-2-P_{aacC1}-*Sputcn32_1291*- *Sputcn32_3328*; Km^r	This study
C*1291*(GGAAF)C*3328*(GGAAF)	CN32 Δ*Sputcn32_1291*Δ*Sputcn32_3328* carrying complement pBBR1MCS-2-P_{aacC1}-*Sputcn32_1291* (GGDEF→GGAAF)-*Sputcn32_3328* (GGEEF→GGAAF); Km^r	This study
Δ*crp*Δ*1291*Δ*3328*C*1291*(GGAAF)C*3328* (GGAAF)	CN32 Δ*crp*Δ*Sputcn32_1291*Δ*Sputcn32_3328* carrying complement pBBR1MCS-2-P_{aacC1}-*Sputcn32_1291* (GGDEF→GGAAF)-*Sputcn32_3328* (GGEEF→GGAAF); Km^r	This study
Δ*1291*Δ*3328*/p*dgcQ*	CN32 Δ*Sputcn32_1291*Δ*Sputcn32_3328* carrying pBBR1MCS-2-P_{aacC1}-*dgcQ*$_{MG1655}$; Km^r	This study
Δ*crp*Δ*1291*Δ*3328*/p*dgcQ*	CN32 Δ*crp*Δ*Sputcn32_1291*Δ*Sputcn32_3328* carrying pBBR1MCS-2-P_{aacC1}-*dgcQ*$_{MG1655}$; Km^r	This study
O*1291*O*3328*	CN32 carrying pBBR1MCS-2-P_{aacC1}-*Sputcn32_1291*- *Sputcn32_3328*;	This study

	Km^r	
Δ*crp*O*1291*O*3328*	CN32 Δ*crp* carrying pBBR1MCS-2-P_{aacC1}-*Sputcn32_1291-Sputcn32_3328*; Km^r	This study
Δ*1291*Δ*3328*Δ*bpfA*	CN32 Δ*Sputcn32_1291*Δ*Sputcn32_3328*Δ*bpfA*	This study
Δ*crp*Δ*1291*Δ*3328*Δ*bpfA*	CN32 Δ*crp*Δ*Sputcn32_1291*Δ*Sputcn32_3328*Δ*bpfA*	This study
Δ*1291*Δ*3328*Δ*bpfD*	CN32 Δ*Sputcn32_1291*Δ*Sputcn32_3328*Δ*bpfD*	This study
Δ*crp*Δ*1291*Δ*3328*Δ*bpfD*	CN32 Δ*crp*Δ*Sputcn32_1291*Δ*Sputcn32_3328*Δ*bpfD*	This study
Δ*1291*Δ*3328*Δ*bpfG*	CN32 Δ*Sputcn32_1291*Δ*Sputcn32_3328*Δ*bpfG*	This study
Δ*crp*Δ*1291*Δ*3328*Δ*bpfG*	CN32 Δ*crp*Δ*Sputcn32_1291*Δ*Sputcn32_3328*Δ*bpfG*	This study
Δ*bpfA*/p*dgcQ*	CN32 Δ*bpfA* carrying pBBR1MCS-2-P_{aacC1}-*dgcQ*$_{\mathrm{MG1655}}$; Km^r	This study
Δ*crp*Δ*bpfA*/p*dgcQ*	CN32 Δ*crp*Δ*bpfA* carrying pBBR1MCS-2-P_{aacC1}-*dgcQ*$_{\mathrm{MG1655}}$; Km^r	This study
Δ*1291*Δ*3328*Δ*bpfA*/p*dgcQ*	CN32 Δ*Sputcn32_1291*Δ*Sputcn32_3328*Δ*bpfA* carrying pBBR1MCS-2-P_{aacC1}-*dgcQ*$_{\mathrm{MG1655}}$; Km^r	This study
Δ*crp*Δ*1291*Δ*3328*Δ*bpfA*/p*dgcQ*	CN32 Δ*crp*Δ*Sputcn32_1291*Δ*Sputcn32_3328*Δ*bpfA* carrying pBBR1MCS-2-P_{aacC1}-*dgcQ*$_{\mathrm{MG1655}}$; Km^r	This study
Δ*bpfA*O*1291*O*3328*	CN32 Δ*bpfA* carrying pBBR1MCS-2-P_{aacC1} -*Sputcn32_1291-Sputcn32_3328*; Km^r	This study
Δ*crp*Δ*bpfA*O*1291*O*3328*	CN32 Δ*crp*Δ*bpfA* carrying pBBR1MCS-2-P_{aacC1}-*Sputcn32_1291-Sputcn32_3328*; Km^r	This study
WT/P_{aacC1}-*bpfA*	CN32 replacing the promoter region of *bpfA* operon with *aacC1* promoter	This study
Δ*crp*/P_{aacC1}-*bpfA*	Δ*crp* replacing the promoter region of *bpfA* operon with *aacC1* promoter	This study
Δ*cya*/P_{aacC1}-*bpfA*	CN32 Δ*cyaA*Δ*cyaB*Δ*cyaC* replacing the promoter region of *bpfA* operon with *aacC1* promoter	This study
WT/P_{aacC1}-*bpfA*/p*dgcQ*	CN32 replacing the promoter region of *bpfA* operon with *aacC1* promoter and carrying pBBR1MCS-2-P_{aacC1}-*dgcQ*$_{\mathrm{MG1655}}$; Km^r	This study
Δ*cya*/P_{aacC1}-*bpfA*/p*dgcQ*	CN32 Δ*cyaA*Δ*cyaB*Δ*cyaC* replacing the promoter region of *bpfA* operon with *aacC1* promoter and carrying pBBR1MCS-2-P_{aacC1}-*dgcQ*$_{\mathrm{MG1655}}$; Km^r	This study
Δ*1291*Δ*3328*/P_{aacC1}-*bpfA*/p*dgcQ*	CN32 Δ*Sputcn32_1291*Δ*Sputcn32_3328* replacing the promoter region of *bpfA* operon with *aacC1* promoter and carrying pBBR1MCS-2-P_{aacC1}-*dgcQ*$_{\mathrm{MG1655}}$; Km^r	This study
Δ*crp*Δ*1291*Δ*3328*/P_{aacC1}-*bpfA*/p*dgcQ*	CN32 Δ*crp*Δ*Sputcn32_1291*Δ*Sputcn32_3328* replacing the promoter region of *bpfA* operon with *aacC1* promoter and carrying pBBR1MCS-2-P_{aacC1}-*dgcQ*$_{\mathrm{MG1655}}$; Km^r	This study
O*1291*O*3328*/P_{aacC1}-*bpfA*	CN32 replacing the promoter region of *bpfA* operon with *aacC1* promoter and carrying pBBR1MCS-2-P_{aacC1}- *Sputcn32_1291-Sputcn32_3328*; Km^r	This study
Δ*crp*O*1291*O*3328*/P_{aacC1}-*bpfA*	CN32 Δ*crp* replacing the promoter region of *bpfA* operon with *aacC1* promoter and carrying pBBR1MCS-2-P_{aacC1}-*Sputcn32_1291-Sputcn32_3328*; Km^r	This study
WT/BpfA-Flag	CN32 BpfA with 3×Flag inserted after residue 3700 aa (11100 bp) in the full-length protein of 4220 aa transformant	This study
Δ*crp*/BpfA-Flag	CN32 Δ*crp* BpfA with 3×Flag inserted after residue 3700 aa (11100 bp) in the full-length protein of 4220 aa transformant	This study
Δ*bpfG*/BpfA-Flag	CN32 Δ*bpfG* BpfA with 3×Flag inserted after residue 3700 aa (11100 bp) in the full-length protein of 4220 aa	This study
Δ*crp*Δ*bpfG*/BpfA-Flag	CN32 Δ*crp*Δ*bpfG* BpfA with 3×Flag inserted after residue 3700 aa (11100 bp) in the full-length protein of 4220 aa transformant	This study
WT/P_{aacC1}-*bpfA*/BpfA-Flag	CN32 replacing the promoter region of *bpfA* operon with *aacC1* promoter	This study

	and BpfA with 3×Flag inserted after residue 3700 aa (11100 bp) in the full-length protein of 4220 aa transformant	
Δ*crp*/P_{aacC1}-*bpfA*/BpfA-Flag	CN32 Δ*crp* replacing the promoter region of *bpfA* operon with *aacC1* promoter and BpfA with 3×Flag inserted after residue 3700 aa (11100 bp) in the full-length protein of 4220 aa transformant	This study
C*crp*/P_{aacC1}-*bpfA*/BpfA-Flag	CN32 Δ*crp* replacing the promoter region of *bpfA* operon with *aacC1* promoter and BpfA with 3×Flag inserted after residue 3700 aa (11100 bp) in the full-length protein of 4220 aa transformant carrying complement pBBR1MCS-2-P_{aacC1}-*crp*; Km^r	This study
C*crp*-R84L/P_{aacC1}-*bpfA*/BpfA-Flag	CN32 Δ*crp* replacing the promoter region of *bpfA* operon with *aacC1* promoter and BpfA with 3×Flag inserted after residue 3700 aa (11100 bp) in the full-length protein of 4220 aa transformant carrying complement pBBR1MCS-2-P_{aacC1}-*crp*-R84L; Km^r	This study
Δ*cya*/P_{aacC1}-*bpfA*/BpfA-Flag	CN32 Δ*cyaA*Δ*cyaB*Δ*cyaC* replacing the promoter region of *bpfA* operon with *aacC1* promoter and BpfA with 3×Flag inserted after residue 3700 aa (11100 bp) in the full-length protein of 4220 aa transformant	This study
Δ*1291*Δ*3328*/P_{aacC1}-*bpfA*/BpfA-Flag	CN32 Δ*1291*Δ*3328* replacing the promoter region of *bpfA* operon with *aacC1* promoter and BpfA with 3×Flag inserted after residue 3700 aa (11100 bp) in the full-length protein of 4220 aa transformant	This study
Δ*crp*Δ*1291*Δ*3328*/P_{aacC1}-*bpfA*/BpfA-Flag	CN32 Δ*crp*Δ*1291*Δ*3328* replacing the promoter region of *bpfA* operon with *aacC1* promoter and BpfA with 3×Flag inserted after residue 3700 aa (11100 bp) in the full-length protein of 4220 aa transformant	This study
O*1291*O*3328*/P_{aacC1}-*bpfA*/BpfA-Flag	CN32 replacing the promoter region of *bpfA* operon with *aacC1* promoter and BpfA with 3×Flag inserted after residue 3700 aa (11100 bp) in the full-length protein of 4220 aa and carrying pBBR1MCS-2-P_{aacC1}-*Sputcn32_1291-Sputcn32_3328* transformant; Km^r	This study
Δ*crp*O*1291*O*3328*/P_{aacC1}-*bpfA*/BpfA-Flag	CN32 Δ*crp* replacing the promoter region of *bpfA* operon with *aacC1* promoter and BpfA with 3×Flag inserted after residue 3700 aa (11100 bp) in the full-length protein of 4220 aa and carrying pBBR1MCS-2-P_{aacC1}-*Sputcn32_1291-Sputcn32_3328* transformant; Km^r	This study
Δ*1291*Δ*3328*/P_{aacC1}-*bpfA*/BpfA-Flag/p*dgcQ*	CN32 Δ*1291*Δ*3328* replacing the promoter region of *bpfA* operon with *aacC1* promoter and BpfA with 3×Flag inserted after residue 3700 aa (11100 bp) in the full-length protein of 4220 aa and carrying pBBR1MCS-2-P_{aacC1}-*dgcQ*$_{MG1655}$ transformant; Km^r	This study
Δ*crp*Δ*1291*Δ*3328*/P_{aacC1}-*bpfA*/BpfA-Flag/p*dgcQ*	CN32 Δ*crp*Δ*1291*Δ*3328* replacing the promoter region of *bpfA* operon with *aacC1* promoter and BpfA with 3×Flag inserted after residue 3700 aa (11100 bp) in the full-length protein of 4220 aa and carrying pBBR1MCS-2-P_{aacC1}-*dgcQ*$_{MG1655}$ transformant; Km^r	This study
WT/P_{aacC1}-*bpfA*/BpfD-Flag/BpfG-HA	CN32 replacing the promoter region of *bpfA* operon with *aacC1* promoter, and with C-terminal 3×Flag-tagged BpfD, and BpfG with 1×HA inserted after residue 221 aa (663 bp) in the full-length protein of 235 aa transformant	This study
Δ*crp*/P_{aacC1}-*bpfA*/BpfD-Flag/BpfG-HA	CN32 Δ*crp* replacing the promoter region of *bpfA* operon with *aacC1* promoter, and with C-terminal 3×Flag-tagged BpfD, and BpfG with 1×HA inserted after residue 221 aa (663 bp) in the full-length protein of 235 aa transformant	This study
C*crp*/P_{aacC1}-*bpfA*/BpfD-Flag/BpfG-HA	CN32 Δ*crp* replacing the promoter region of *bpfA* operon with *aacC1*	This study

	promoter, and with C-terminal 3×Flag-tagged BpfD, and BpfG with 1×HA inserted after residue 221 aa (663 bp) in the full-length protein of 235 aa transformant carrying complement pBBR1MCS-2-P$_{aacC1}$-*crp*; Km^r	
C*crp*-R84L/P$_{aacC1}$-*bpfA*/BpfD-Flag/BpfG-HA	CN32 Δ*crp* replacing the promoter region of *bpfA* operon with *aacC1* promoter, and with C-terminal 3×Flag-tagged BpfD, and BpfG with 1×HA inserted after residue 221 aa (663 bp) in the full-length protein of 235 aa transformant carrying complement pBBR1MCS-2-P$_{aacC1}$-*crp*-R84L; Km^r	This study
Δ*cya*/P$_{aacC1}$-*bpfA*/BpfD-Flag/BpfG-HA	CN32 Δ*cyaA*Δ*cyaB*Δ*cyaC* replacing the promoter region of *bpfA* operon with *aacC1* promoter, and with C-terminal 3×Flag-tagged BpfD, and BpfG with 1×HA inserted after residue 221 aa (663 bp) in the full-length protein of 235 aa transformant	This study
Δ*1291*Δ*3328*/P$_{aacC1}$-*bpfA*/BpfD-Flag/BpfG-HA	CN32 Δ*1291*Δ*3328* replacing the promoter region of *bpfA* operon with *aacC1* promoter, and with C-terminal 3×Flag-tagged BpfD, and BpfG with 1×HA inserted after residue 221 aa (663 bp) in the full-length protein of 235 aa transformant	This study
Δ*crp*O*1291*O*3328*/P$_{aacC1}$-*bpfA*/BpfD-Flag/BpfG-HA	CN32 Δ*crp* replacing the promoter region of *bpfA* operon with *aacC1* promoter, and with C-terminal 3×Flag-tagged BpfD, and BpfG with 1×HA inserted after residue 221 aa (663 bp) in the full-length protein of 235 aa, and carrying pBBR1MCS-2-P$_{aacC1}$- *Sputcn32_1291-Sputcn32_3328* transformant; Km^r	This study
WT/P$_{aacC1}$-*bpfA*/BpfD-Flag/BpfG-HA/CRP-His	CN32 replacing the promoter region of *bpfA* operon with *aacC1* promoter, and with C-terminal 3×Flag-tagged BpfD, and BpfG with 1×HA inserted after residue 221 aa (663 bp) in the full-length protein of 235 aa, and with C-terminal 10×His-tagged CRP transformant.	This study
Δ*cya*/P$_{aacC1}$-*bpfA*/BpfD-Flag/BpfG-HA/CRP-His	CN32 Δ*cyaA*Δ*cyaB*Δ*cyaC* replacing the promoter region of *bpfA* operon with *aacC1* promoter, and with C-terminal 3×Flag-tagged BpfD, and BpfG with 1×HA inserted after residue 221 aa (663 bp) in the full-length protein of 235 aa, and with C-terminal 10×His-tagged CRP transformant.	This study
WT/P$_{aacC1}$-*bpfA*/BpfD-Flag/BpfG-HA/CRP-R84L-His	CN32 replacing the promoter region of *bpfA* operon with *aacC1* promoter, and with C-terminal 3×Flag-tagged BpfD, and BpfG with 1×HA inserted after residue 221 aa (663 bp) in the full-length protein of 235 aa, and with C-terminal 10×His-tagged site-directed CRP-R84L transformant.	This study
Plasmids		
pET-28a(+)	Vector for heterologous protein expression in *E. coli*; Km^r	Novagen
pK19*mobsacB*	Suicide plasmid for CN32; *sacB* (modified from *B. subtilis*) *lacZ*; Km^r	74
pRL27	Plasmid containing Mini-Tn5 transposon and a *pir* protein-dependent DNA replication origin (*ori*R6K); Km^r	73
pBBR1MCS-2	Broad-host-range vector; Km^r	76
pMAL-c2x	Vector for heterologous protein expression in *E. coli*; Amp^r	Lab stock
pGEX-4T-1	Vector for heterologous protein expression in *E. coli*; Amp^r	Lab stock
pRK2013	Helper plasmid in matings; Km^r	75

Supplementary Table 3 Primers used in this study.

Primer	Sequence (5' to 3')	Usage
Tn5-seq1	AACAAGCCAGGGATGTAACG	Mini-Tn5 sequence
Tn5-seq2	CAGCAACACCTTCTTCACGA	Mini-Tn5 sequence
crp-5F	CTCAAAGAATTCTAAGATGAGTCCAATCACTGTGCCC	*crp* knock-out
crp-5R	GTATACTCTAGAGGTACCCGTTAAGTTAGTCTTCAGC	*crp* knock-out
crp-3F	TTTTACTCTAGAGATGTAATAAAGGGTATCTGAATCT	*crp* knock-out
crp-3R	AGGCAGAAGCTTCAGCGAGGTTATCTAAATTAGTGGG	*crp* knock-out
crp-UF	GTTGGATACACCAGTGCGAACAGAC	*crp* knock-out
crp-DR	TCTAAACTAAGACTTCTATCAAGTT	*crp* knock-out
crp-INF	CCAATCTCTTGACGAGTGATCTTG	*crp* knock-out
crp-INR	AAGGTTCTGTTGCCGTATTGATTAA	*crp* knock-out
crp-OF	CCAGCATGATATGTTCAAGATCTT	*crp* knock-out
crp-OR	GCAGCACTAAAATCACCAATTTCT	*crp* knock-out
crp-comF	CGGGATCCAATCAATCGAGGAACATCAACATGG	*crp* complement
crp-comR	CCCAAGCTTGCTGAAGACTAACTTAACGGGTACC	*crp* complement
cyaA-5F	TTGATTCTGCAGCGACGTTAAAACAGTAAATACCCAG	*cyaA* knock-out
cyaA-5R	GGAATTGGATCCGATGTTACCGACCAAGATGCTGAG	*cyaA* knock-out
cyaA-3F	TTCAAAGGATCCAGCATAGGTATCCATCTCTTGAGGT	*cyaA* knock-out
cyaA-3R	TTTCTAGAATTCAATGCTCTAATGGTGCTAGAAGTGC	*cyaA* knock-out
cyaA-UF	TGTAGATGGCTCTAAGATTGTGATC	*cyaA* knock-out
cyaA-DR	AGGATGGATCAGCAAGACCTATTTC	*cyaA* knock-out
cyaA-INF	AATCCTGAACCAGTTGCTGCATT	*cyaA* knock-out
cyaA-INR	ACCTTGGTGATGTCCATGAGTTAC	*cyaA* knock-out
cyaA-OF	AATGGTTGGATCTCAGTGAGTTGTG	*cyaA* knock-out
cyaA-OR	CTTGGCATCCACCTTCTAAGCGAG	*cyaA* knock-out
cyaA-comF	AGACATGGATCCGCCTAGTTTCCAGTCAAAGATCTTAG	*cyaA* complement
cyaA-comR	TCCCATGAATTCGGTGCAAATTAGTACGATTAAAACT	*cyaA* complement
cyaB-5F	CACTGTCTGCAGGCAATTGGACTTGATGATGTTTGAC	*cyaB* knock-out
cyaB-5R	CTAAAGGGATCCCAAGCACTGAACCTAAGGACATTT	*cyaB* knock-out
cyaB-3F	TGGTGCGGATCCAAGACTTTCAGGTATCACACTATTC	*cyaB* knock-out
cyaB-3R	CTTATTGAATTCATGATGCTGTATTTAGCTGCCAATG	*cyaB* knock-out
cyaB-UF	CGCAATACACAGCAACACGGAGTAT	*cyaB* knock-out
cyaB-UR	ATGCACCACTGTTATTGTTAGATG	*cyaB* knock-out
cyaB-INF	CATACATGGCTAATAAGGATTCATC	*cyaB* knock-out
cyaB-INR	GATAACCTTGAGCATGATTGTTAC	*cyaB* knock-out
cyaB-OF	TTTCAAAGTGATACGAGGGGATTG	*cyaB* knock-out
cyaB-OR	CCAAGCCATGTTGATCCTCTCCAC	*cyaB* knock-out
cyaB-comF	GGTTAAGAATTCTCACCTTTAGTCGGCCGTTATTTG	*cyaB* complement
cyaB-comR	AGGATTAAGCTTTGATTTTATCCTCGCAAGCATCCT	*cyaB* complement
cyaC-5F	AAATTTCTGCAGAGAAGAGCAACTAGGTCATCAATTG	*cyaC* knock-out
cyaC-5R	TCCCCAGGATCCCTCTGATCTTGACTCAATTTAAGCT	*cyaC* knock-out
cyaC-3F	CTGGGGGGATCCCAGTTCTGGTGATTGTGCATATCG	*cyaC* knock-out

cyaC-3R	ATCCATGAATTCAATATCTGTGGATAAGTATGTGAGG	*cyaC* knock-out
cyaC-UF	AGGCTCCTTACCGAGATATAGGAC	*cyaC* knock-out
cyaC-DR	CACTAGCACTTTGGTGATGATTTTC	*cyaC* knock-out
cyaC-INF	TAAGGACGGCATCACCCATGTAAC	*cyaC* knock-out
cyaC-INR	AAACCATATGTCTACGCTAAGGCA	*cyaC* knock-out
cyaC-OF	CTGAATGGCGATTCTGCTCACACTC	*cyaC* knock-out
cyaC-OR	TTTCATTAGGTTAAAGGCATTCAC	*cyaC* knock-out
cyaC-comF	AAATTTAAGCTTCACTAGCACTTTGGTGATGATTTTC	*cyaC* complement
cyaC-comR	AGTTACCTCGAGAGCTTAAATTGAGTCAAGATCAGAG	*cyaC* complement
cpdA-5F	ATGACACTGCAGTTCAGCGCGATCAAGTTGTGTTAA	*cpdA* knock-out
cpdA-5R	GACCGCGGATCCCCGAATGGTATTAAGCACAGCAGC	*cpdA* knock-out
cpdA-3F	GATAATGGATCCGATTCCAGCGGTTATTAACGCGAG	*cpdA* knock-out
cpdA-3R	TGACCAGAATTCTCACGACGCACTGTAACTTCAACA	*cpdA* knock-out
cpdA-UF	GATTTACCAATTACGCCCAGTGTA	*cpdA* knock-out
cpdA-DR	CCGTGAATATAGAGCAGCATAGTT	*cpdA* knock-out
cpdA-INF	ATCCGCGTATTATGTATCTGCATA	*cpdA* knock-out
cpdA-INR	ATAGTGAGTATCAATTCGCTGATG	*cpdA* knock-out
cpdA-OF	GTGTCAAATTGCCTTGATAATAGG	*cpdA* knock-out
cpdA-OR	GTTCCTGTGACTTTTAACTCTTCA	*cpdA* knock-out
cpdA-comF	TAGGGATCCAATTTGGGTATTCAATGTGCTGAA	*cpdA* complement
cpdA-comR	TGCGGTCACTCGCGTTAATAAC	*cpdA* complement
bpfA-5F	TTAATGCTGCAGAACTCAATATAGTTACCCATTGCG	*bpfA* knock-out
bpfA-5R	ATCATCGGATCCGAAGGTGATTTCATATGTTGCATTT	*bpfA* knock-out
bpfA-3F	CATAACGGATCCAATGGCACTATGATCCCTTAAATA	*bpfA* knock-out
bpfA-3R	TCAATTGAATTCAAATGTCCATGTTTAAGGCTTGAA	*bpfA* knock-out
bpfA-UF	AAGCCTTAATACCAAGCGATAGAG	*bpfA* knock-out
bpfA-DR	AATGGATCAACTATAACTCGCTGC	*bpfA* knock-out
bpfA-INF	ACCAATGGCAATGTCTTTAGCATC	*bpfA* knock-out
bpfA-INR	TGTAGGTAAAGCTGTCACTGCCAT	*bpfA* knock-out
bpfA-OF	TTAGCACGCGTTGAGCTCGATAA	*bpfA* knock-out
bpfA-OR	AAATGACATTATCAGCAACCCGAT	*bpfA* knock-out
bpfA-QF	ACCATCGCACCCAATGAGTT	*bpfA* qPCR
bpfA-QR	CTGCATCGCTGTCATTGTCG	*bpfA* qPCR
bpfA-EMSA-F	TACAAATAAACATACAACATCTAGT	*bpfA* EMSA probe
bpfA-EMSA-R	ACCGATCCCATAATTAGC	*bpfA* EMSA probe
bpfD-5F	TCGTTTCTGCAGTGTTGATGATATAAAACTGTGGGG	*bpfD* knock-out
bpfD-5R	GTTGCTGGATCCTTAGACGTTGTTTAGATCGGACTCCAT	*bpfD* knock-out
bpfD-3F	AAAGTAGGATCCTTAGCGATCCCCAATTTCATGAGT	*bpfD* knock-out
bpfD-3R	AGGTTCGAATTCGAATCCTCTATCACCGACCAAATA	*bpfD* knock-out
bpfD-UF	GCTTAGGTTATGTGCAATTTACTG	*bpfD* knock-out
bpfD-DR	TGCCAGTCACTATCACTTGAATAT	*bpfD* knock-out
bpfD-INF	TCAGTGGTTTATCAATCTCAATCT	*bpfD* knock-out
bpfD-INR	CAAGATCCTTCAGTTCAGTGGTTC	*bpfD* knock-out
bpfD-OF	CTCAACTCTGGTTAAACATTATGG	*bpfD* knock-out

bpfD-OR	AAGGTAATACAGATTGACGGTGAT	*bpfD* knock-out
bpfG-5F	CAAAAGGAATTCATACTCAATCTGCCTATGCGCAAC	*bpfG* knock-out
bpfG-5R	ACGTTCTCTAGAGCCATAATGTTTAACCAGAGTTGAG	*bpfG* knock-out
bpfG-3F	AGGCAATCTAGACCTAAATTAAAACTGGAGTAGCCC	*bpfG* knock-out
bpfG-3R	TGATTTCTGCAGGATCGTGCTCAACATCTGTGACTA	*bpfG* knock-out
bpfG-UF	GACAATTTACTCTGTCGATGACAA	*bpfG* knock-out
bpfG-DR	CACATAACCTAAGCTTGCGACTAC	*bpfG* knock-out
bpfG-INF	ATCTGAAGCAAGTACGTTAGATGA	*bpfG* knock-out
bpfG-INR	ACGGTCAACACCTAATCTGTGGTT	*bpfG* knock-out
bpfG-OF	CTTTTCGCTGGAGGAAGAGATCTT	*bpfG* knock-out
bpfG-OR	TCTTGGTTAATTGTACGGATCAATG	*bpfG* knock-out
dgcQ-comF	CCAGAAGGATCCTCATAAAAAAGCAGGTTGGGAGTCGTCAG	*dgcQ*$_{MG1655}$ amplification
dgcQ-comR	CGCCCGGAATTCTTAAGCGTTATCGCTCGCGAATACC	*dgcQ*$_{MG1655}$ amplification
0133-QF	ATGGCTACGCGCATTATCGAAACC	*Sputcn32_0133* qPCR
0133-QR	CACATCAAGGCCATCCTCTGGGTA	*Sputcn32_0133* qPCR
0133-5F	CTTCGGGAATTCTGTCTGACACAGGACTCACTTGTT	*Sputcn32_0133* knock-out
0133-5R	GAAGCTGGATCCGAATGCAGTAATTTCTAAGCGTAAA	*Sputcn32_0133* knock-out
0133-3F	TGTTTGGGATCCTATTGCTAGATGACTATGATCCGC	*Sputcn32_0133* knock-out
0133-3R	GCGTTTCTGCAGACTGTGAGCATTGAGGCAAAAGTC	*Sputcn32_0133* knock-out
0133-UF	AGTTGACCTATGAACATGAACAGT	*Sputcn32_0133* knock-out
0133-DR	TGTATGCCATCAACGCTACATCAT	*Sputcn32_0133* knock-out
0133-INF	GAATCGAGTCGTATCTGGTACTTT	*Sputcn32_0133* knock-out
0133-INR	TTGAGATCACTGAAAGTATGCTGA	*Sputcn32_0133* knock-out
0133-OF	TTTGTGTATTTAAGTCGGCAAAGC	*Sputcn32_0133* knock-out
0133-OR	AACAACTCAAGCCAATATGGTACT	*Sputcn32_0133* knock-out
0654-QF	TGGCAGCAGTTGGATCTTGTTTCC	*Sputcn32_0654* qPCR
0654-QR	AAGCGCATTTCAAGCCAGTTCATA	*Sputcn32_0654* qPCR
0654-5F	TGGGCGGAATTCCTATGTGGAACAAGAGGTGCCATC	*Sputcn32_0654* knock-out
0654-5R	TTGTAAACTAGTGGTAATAGTCTCTAGGGCTCGGTC	*Sputcn32_0654* knock-out
0654-3F	TGGCTTACTAGTGATATGAACAGTGGTTTACAGCCT	*Sputcn32_0654* knock-out
0654-3R	AATCGTCTGCAGTAAGCTCGATTCTGCTACGTTGGT	*Sputcn32_0654* knock-out
0654-UF	CTTTCCTTATCAGTCCTGCTTATT	*Sputcn32_0654* knock-out
0654-DR	AATCTCTTGCCCTACGTTCTGTTG	*Sputcn32_0654* knock-out
0654-INF	TCTGAGTGTTGTGATTGGAGTAAT	*Sputcn32_0654* knock-out
0654-INR	TCAGCTATCATCACCAGCGTATTT	*Sputcn32_0654* knock-out
0654-OF	ATGGCAGTGCTGATGCTCAATTTT	*Sputcn32_0654* knock-out
0654-OR	TTCTTGACTCCATAATCGAATACA	*Sputcn32_0654* knock-out
1291-QF	AGCGGATCGATGAGGCATTACCAG	*Sputcn32_1291* qPCR
1291-QR	GCTAATCCCGCTCGACTTTCTTCG	*Sputcn32_1291* qPCR
1291-5F	ACCTCTCTGCAGGCGGGATTATCAATGATAATTTGA	*Sputcn32_1291* knock-out
1291-5R	AGGGTTGGATCCGTGATTGACGTATAGTTAAGACCG	*Sputcn32_1291* knock-out
1291-3F	AATAAAGGATCCTTGGAGTAGTGTTTGTAACTTGGC	*Sputcn32_1291* knock-out
1291-3R	GAGCAGGAATTCCAACTCAAGCTTGAACTTAGCCTG	*Sputcn32_1291* knock-out
1291-UF	GTGAGCAGTTACGATACAGTTGAT	*Sputcn32_1291* knock-out

1291-DR	ATCGACTTCACAGCTAATCTGGAC	*Sputcn32_1291* knock-out
1291-INF	CTTCGGTACTGAGTTCATTGAGTT	*Sputcn32_1291* knock-out
1291-INR	ATTACAGCAGCAATTTAGCGAATC	*Sputcn32_1291* knock-out
1291-OF	TGTTCCATACCTTGTTTCATTACC	*Sputcn32_1291* knock-out
1291-OR	AGCAGATCATTGAATTAAGTTCAC	*Sputcn32_1291* knock-out
1291-comF	CACAGCTCTAGATAATCTGGACATAGGATCGTCATG	*Sputcn32_1291* complement
1291-comR	GCGTATGGATCCAGTCGGTCTTAACTATACGTCAAT	*Sputcn32_1291* complement
1291-GGAAF-F[a]	GCTACGGCGGTGCGGCGTTTGTCCTCTTAC	*Sputcn32_1291* site mutagenesis
1291-GGAAF-R[a]	GTGCAACAAATTCTGTATCTCGTAGGGAGT	*Sputcn32_1291* site mutagenesis
1365-QF	TTACTCACGCCACTGACACCGTCA	*Sputcn32_1365* qPCR
1365-QR	CAAACATCGCCTGCGTTGACATTT	*Sputcn32_1365* qPCR
1365-5F	GGGCATGAATTCTCAAGTGCGACCTTTATTGGATGT	*Sputcn32_1365* knock-out
1365-5R	AAGGCAGGATCCTCAGGTCGCAATGGTTATAAACTA	*Sputcn32_1365* knock-out
1365-3F	GCAGGCGGATCCAAAGACTGTCCGAGGATCTAAACT	*Sputcn32_1365* knock-out
1365-3R	ATAAGCCTGCAGCGAAGCCAATACACCTCATCCATA	*Sputcn32_1365* knock-out
1365-UF	TTTGTGTAGAGGATGAGGCTGTTG	*Sputcn32_1365* knock-out
1365-DR	GAACTTACAACTTAACCAACTGAT	*Sputcn32_1365* knock-out
1365-INF	TCAGCCTCTAGAATATCGTTAGAC	*Sputcn32_1365* knock-out
1365-INR	TTGGCCATAAATGCGGTGACTATA	*Sputcn32_1365* knock-out
1365-OF	GACAGATCCAGCCGTTAATGCAGG	*Sputcn32_1365* knock-out
1365-OR	AGAATAACACCGACACAATTGAGC	*Sputcn32_1365* knock-out
1412-QF	ATTCCGAGCCATGAGCCATTGTTC	*Sputcn32_1412* qPCR
1412-QR	GGTTCGCAAGTCCCGTTAGTGAGT	*Sputcn32_1412* qPCR
1412-5F	TTCATCCTGCAGTACGGCATTTATATGGACAGTGGA	*Sputcn32_1412* knock-out
1412-5R	GATGGTGGATCCCGCGACCGAATTAATACATCACTT	*Sputcn32_1412* knock-out
1412-3F	AATAAAGGATCCGAGTTCAATGGTCTGGAGGGAAGA	*Sputcn32_1412* knock-out
1412-3R	CCCTTCGAATTCTATCATGTGATAAATCGCTGTGTC	*Sputcn32_1412* knock-out
1412-UF	ACTGGCATACTGGTAAATATGGCC	*Sputcn32_1412* knock-out
1412-UR	ACAATCCGCTTTATCCATTGTCGA	*Sputcn32_1412* knock-out
1412-INF	GCACTGACAGAGACATAGTCACGT	*Sputcn32_1412* knock-out
1412-INR	GATGAAATACTGCAACACTATTGG	*Sputcn32_1412* knock-out
1412-OF	TTTGCTTGAGTAATGACGGTATTT	*Sputcn32_1412* knock-out
1412-OR	GAATGCTTATCGATGCTAATACAA	*Sputcn32_1412* knock-out
1858-QF	TGGCCGATCAGCTTGCATTAACCC	*Sputcn32_1858* qPCR
1858-QR	CTGTTGCACTGCGAAATAGGCTTA	*Sputcn32_1858* qPCR
1858-5F	CGATGGGAATTCCTGAAGCTCTGAGTAATTGTGATT	*Sputcn32_1858* knock-out
1858-5R	TTTAGGGGATCCTCTATGGATATCTTGCAACATCAT	*Sputcn32_1858* knock-out
1858-3F	AATCGTGGATCCTCTTCTGTACTGACACATTGAATG	*Sputcn32_1858* knock-out
1858-3R	GCGCCACTGCAGTAGATACTGGCTATTATCTAGTGC	*Sputcn32_1858* knock-out
1858-UF	AAAGTCAATCTCTCGTAGCACAAT	*Sputcn32_1858* knock-out
1858-DR	TCGATGCAATCTATGAACATTCAG	*Sputcn32_1858* knock-out
1858-INF	AATGGACATCTTGAAGGTGTTCTC	*Sputcn32_1858* knock-out
1858-INR	ACTCTCGCAAATGACTACCAATGC	*Sputcn32_1858* knock-out
1858-OF	TTATCCTTATTTGAACCTGTTGTT	*Sputcn32_1858* knock-out

1858-OR	CGTTTGAATCTGTAGATCGATTAA	*Sputcn32_1858* knock-out
1934-QF	GTTTATCCAGCAGACTGAACGGCG	*Sputcn32_1934* qPCR
1934-QR	GGGTAAGCCATCAACACCGAGATC	*Sputcn32_1934* qPCR
1934-5F	AGTTAAGAATTCAGCATTGGAACCAGAGACTAAAGT	*Sputcn32_1934* knock-out
1934-5R	CGGTTCGGATCCTCCTTCGCTATCCACATATTGATA	*Sputcn32_1934* knock-out
1934-3F	AGTGGGGGATCCGGACGATGTTACCGTATTTCTTGA	*Sputcn32_1934* knock-out
1934-3R	CGACGCCTGCAGATGGCTTCGTTATAGGTCATTCGC	*Sputcn32_1934* knock-out
1934-UF	CTTAATTGTGATCTTGTGCAGCTA	*Sputcn32_1934* knock-out
1934-DR	CAATAATGACTGCGCATCTAACTC	*Sputcn32_1934* knock-out
1934-INF	ATTGCTGATGTTGATGCTTATCTT	*Sputcn32_1934* knock-out
1934-INR	TAAGCCATCAACACCGAGATCAAC	*Sputcn32_1934* knock-out
1934-OF	CAATGCTGTAGATTTGAATCGGCG	*Sputcn32_1934* knock-out
1934-OR	AAGTAAATCTGCAATGTCCACCAG	*Sputcn32_1934* knock-out
3319-QF	AATCATGCCGTTAGCGAGGTCATG	*Sputcn32_3319* qPCR
3319-QR	TTGCTGTGCCACGGTATTTGCACT	*Sputcn32_3319* qPCR
3319-5F	GTAAAGGAATTCCGTCATACTCTCAACAGTTTGCTT	*Sputcn32_3319* knock-out
3319-5R	ACAGAGGGATCCGGTTTCGAGTTCACTGAGTTTACC	*Sputcn32_3319* knock-out
3319-3F	AATGCAGGATCCCTCAATCCACACGCATTAATCGCT	*Sputcn32_3319* knock-out
3319-3R	GTCTGGCTGCAGGGAGATAAACCAAATCGCACTGGC	*Sputcn32_3319* knock-out
3319-UF	TTCTAAGTCATAGACTCCGCAAAC	*Sputcn32_3319* knock-out
3319-DR	GTTAGAGAATGATAAAGTGATCGT	*Sputcn32_3319* knock-out
3319-INF	GATATTCTGGCTTAAGACTCTGGT	*Sputcn32_3319* knock-out
3319-INR	CATTGGCTTCTATCTGCAGTACCT	*Sputcn32_3319* knock-out
3319-OF	ACCATAATCAGTGCTATAACCTAA	*Sputcn32_3319* knock-out
3319-OR	ACATTATCATAGAGAGAAGATTGC	*Sputcn32_3319* knock-out
3328-QF	TTGGTGCCAGTATTGGCGTTGCTT	*Sputcn32_3328* qPCR
3328-QR	CTTGGCGCGGTACATCGCAATATC	*Sputcn32_3328* qPCR
3328-5F	CTCGATGAATTCATATTCAACTATCGGTTCAGTGAC	*Sputcn32_3328* knock-out
3328-5R	GAGTTTGGATCCTGTACGGCTCGCAATTGTAATATT	*Sputcn32_3328* knock-out
3328-3F	TACGGTGGATCCTTGGTCTTGAGTCATCGCATTGAG	*Sputcn32_3328* knock-out
3328-3R	GATGCGCTGCAGCGGCAGTTAATTGAGGATATGTTT	*Sputcn32_3328* knock-out
3328-UF	TGCATGGATTCGAGGAATGATTGC	*Sputcn32_3328* knock-out
3328-DR	TAATCGATGATGATGAAGTGGATA	*Sputcn32_3328* knock-out
3328-INF	AATACTTGTAATCATCATCCGTCA	*Sputcn32_3328* knock-out
3328-INR	AACTGGGTGCTCAGGATTTCTTAC	*Sputcn32_3328* knock-out
3328-OF	ATCCATTAAGCAATGATTGGCCTA	*Sputcn32_3328* knock-out
3328-OR	GCTACTCGATGGATTATTAATGTT	*Sputcn32_3328* knock-out
3328-comF	AAGGTTGGATCCACTGGCGTATTGTTGAGCTTCCAT	*Sputcn32_3328* complement
3328-comR	TACAGGCTCGAGAATGACCACTGGATAAGGTTCACA	*Sputcn32_3328* complement
3328-GGAAF-F[a]	TTTAGGGGGCGCGGCGTTTGTTGTCCTGGT	*Sputcn32_3328* site mutagenesis
3328-GGAAF-R[a]	CGAGCAATGACATCACCATCCCGCAGAACC	*Sputcn32_3328* site mutagenesis
3598-QF	AGAGCAAGCGATACAACAGCGCAC	*Sputcn32_3598* qPCR
3598-QR	TTGTAGACGTTCAGCTCTGCGCCT	*Sputcn32_3598* qPCR
3598-5F	TAGCGCCTGCAGCGCATATAGTATGGACAAATAGCC	*Sputcn32_3598* knock-out

3598-5R	GGTTTGGGATCCTTACACGACTAATACACCAGTCATTAT	*Sputcn32_3598* knock-out
3598-3F	GCAACAGGATCCAGATTGTGCTTAAACTAAATGTACGAT	*Sputcn32_3598* knock-out
3598-3R	TACGAAGAATTCGATTATCTATACGAAACTAGCACC	*Sputcn32_3598* knock-out
3598-UF	ACAACTGATCTCCGCTGACAACTT	*Sputcn32_3598* knock-out
3598-DR	GAAGACAATATCGATGAGGAGCGT	*Sputcn32_3598* knock-out
3598-INF	AGAACTTAGCCAAGCCTACGATAA	*Sputcn32_3598* knock-out
3598-INR	AGTTCCTGGATGCTTATTACCTAG	*Sputcn32_3598* knock-out
3598-OF	TACTTATCATGCTAACCTAAAAGC	*Sputcn32_3598* knock-out
3598-OR	GCTATGTCATCTTTGTATCAGTTT	*Sputcn32_3598* knock-out
P_{bpfA}-5F	TTTTATGGATCCGCAGTATTCCAAGTACTTGAATGGT	Replacing P_{bpfA} with P_{aacC1}
P_{bpfA}-3R	GATGGTGAATTCCACGGTAAAGCTGTCGGTGCCATT	Replacing P_{bpfA} with P_{aacC1}
P_{aacC1}-F	GGACTAGTGAATTGACATAAGCCTGTTCGGTTC	Replacing P_{bpfA} with P_{aacC1}
P_{aacC1}-R[a]	CGTTGCTGCTCCATAACATCAAAC	Replacing P_{bpfA} with P_{aacC1}
$P_{bpfA-aacC1}$-KinF[a]	ATGGGATCGGTCATTACATCAAAAAAAGGT	Replacing P_{bpfA} with P_{aacC1}
$P_{bpfA-aacC1}$-KinR	GGACTAGTCTATCGCTTGGTATTAAGGCTTTTTCTTGC	Replacing P_{bpfA} with P_{aacC1}
$P_{bpfA-aacC1}$-SF	TAGCTCCTTATCCTTCCTCATATC	Replacing P_{bpfA} with P_{aacC1}
$P_{bpfA-aacC1}$-SR	CATCGAAGGTGATTTCATATGTTG	Replacing P_{bpfA} with P_{aacC1}
Flag-F	CCGCTCGAGGGAGGTGGCGATTACAAGGATGAC	*flag* amplification
$Flag_{bpfA}$-R	GGGGATCCGCCACCTCCTTTATCGTCATCATCTTTGTAGTC	*flag* amplification
$Flag_{bpfD}$-R	GGGGATCCTTTATCGTCATCATCTTTGTAGTC	*flag* amplification
CRP-C5F	TTTCACGGATCCCTTACTTACCTCACCTCAAAGCGA	CRP C-teminal 10×His knock-in
CRP-C3R	TCAAATGAATTCAAGATGAGTCCAATCACTGTGCCC	CRP C-teminal 10×His knock-in
CRP-CSF	GCAAATCAAGATCACTCGTCAAGA	CRP C-teminal 10×His knock-in
CRP-CSR	ATTATTGTTGGATACACCAGTGCG	CRP C-teminal 10×His knock-in
CRP-COF	CAAAACCCATTGTCTGTATTTCAC	CRP C-teminal 10×His knock-in
CRP-COR	GGCGCAACATGGACTCAAAT	CRP C-teminal 10×His knock-in
CRP-His_{10}KinF[a]	CATCATCATCATCATTAAGTTAGTCTTCAGCTTGATTTAAGCCTG TTTCATT	CRP C-teminal 10×His knock-in
CRP-His_{10}KinR[a]	ATGATGATGATGATGACGGGTACCGTATACCACTATGGTTTTGCC GTGTGCT	CRP C-teminal 10×His knock-in
BpfA-C520-5F	AATTTAGAATTCGGTTCTATAGCCTATGTTGAATTATC	BpfA C-teminal 520 aa 3×Flag knock-in
BpfA-C520-3R	ATTAGCCTGCAGCAATAATAGGCCGTTACTATCAAC	BpfA C-teminal 520 aa 3×Flag knock-in
BpfA-C520-SF	TGATGGTCCAGATAACGATACAGG	BpfA C-teminal 520 aa 3×Flag knock-in
BpfA-C520-SR	ATCCATTACCAGCAACCATACGAA	BpfA C-teminal 520 aa 3×Flag knock-in
BpfA-C520-OF	AAGCCTATGATGAGCAAGGTAATT	BpfA C-teminal 520 aa 3×Flag knock-in
BpfA-C520-OR	TGCCACCATTTACAATATCCGCTT	BpfA C-teminal 520 aa 3×Flag knock-in
BpfA-C520 -FlagKinF	CGGGATCCAACGAAACACCTAATACAAATGACCCAATA	BpfA C-teminal 520 aa 3×Flag knock-in

BpfA-C520 -FlagKinR	CCGCTCGAGATGGCTAAAGTTGAGAGTTAACGTTACAGG	BpfA C-teminal 520 aa 3×Flag knock-in
BpfD-C5F	TATGAGGAATTCCCAGTGGCAATTAACTTAACACAC	BpfD C-teminal 3×Flag knock-in
BpfD-C3R	GGTGATCTGCAGTAGCGCAGTCCGGTAGCGCATCTG	BpfD C-teminal 3×Flag knock-in
BpfD-CSF	ATTACGATGAAGCACAGCATAACA	BpfD C-teminal 3×Flag knock-in
BpfD-CSR	TATAGATAGGGTGGAGAGTCGTAA	BpfD C-teminal 3×Flag knock-in
BpfD-COF	TACATTGAATTATTATCGCTTGGTGC	BpfD C-teminal 3×Flag knock-in
BpfD-COR	GACCAAATTCTTCGCATAACTAGC	BpfD C-teminal 3×Flag knock-in
BpfD-FlagKinF	CGGGATCCTAAATCAGTCGTGAAGCTCCTTCGGGAGCT	BpfD C-teminal 3×Flag knock-in
BpfD-FlagKinR	CCGCTCGAGTTCTAGATTTTCTGGTGGCGCAATAAATCC	BpfD C-teminal 3×Flag knock-in
BpfG-C14-5F	TATGGCGAATTCTCTAGTCGCCCAATAGTATTAGAA	BpfG C-teminal 14 aa HA knock-in
BpfG-C14-3R	ATGTACCTGCAGTTCTTCAAGCCAATCTAGTTTTGC	BpfG C-teminal 14 aa HA knock-in
BpfG-C14-SF	TTAACGGTAAACAACTATGGCTTA	BpfG C-teminal 14 aa HA knock-in
BpfG-C14-SR	CGTTGTTTAGATCGGACTCCATTT	BpfG C-teminal 14 aa HA knock-in
BpfG-C14-OF	CGCTAGCAGATCGTTTTAACATCG	BpfG C-teminal 14 aa HA knock-in
BpfG-C14-OR	CGAGAATACGTATGGTTTCATCTC	BpfG C-teminal 14 aa HA knock-in
BpfG-HAKinF[a]	GTCCCAGACTACGCTGCGGGTGCGTTAGGTGTTGACCGTCTCAGGCAACCTAAA	BpfG C-teminal 14 aa HA knock-in
BpfG-HAKinR[a]	GTCGTATGGGTAGAGGAGATTTTGTCTGTGGTTGAGGTCATTCCATTTTTCGAG	BpfG C-teminal 14 aa HA knock-in
His-CRP-F	ATCAACGGATCCATGGCTCTGATTGGTAAGCCAAAACC	Heterologous expression of CRP protein in *E. coli*
His-CRP-R	TGGCCTAAGCTTAGAATTTATGCTAGGCCACTTTAATG	Heterologous expression of CRP protein in *E. coli*
GST-BpfD-InF	CGGAATTCAATACCATCATCGCTTTCTCCCTATTGTTT	Heterologous expression of BpfD protein in *E. coli*
GST-BpfD-InR	AAGGAAAAAAGCGGCCGCTTATTCTAGATTTTCTGGTGGCGCAATAAA	Heterologous expression of BpfD protein in *E. coli*
His-CRP-R84L-F[a]	AAACAAGCATGTGAAATTGCAGAAATTTCA	Heterologous expression of CRP-R84L protein in *E. coli* or complement CRP-R84L to CN32
His-CRP-R84L-R[a]	AGCTCGAACCCAAGCGGTTAATTCTGCTTG	Heterologous expression of CRP-R84L protein in *E. coli* or complement CRP-R84L to CN32
MBP-1291-F	CTCCGGGGATCCATGAAGGATTCAAACGCAACCGTTT	Heterologous expression of 1291 protein in *E. coli*
MBP-1291-R	GCGTATCTGCAGAGTCGGTCTTAACTATACGTCAAT	Heterologous expression of 1291 protein in *E. coli*

[a] 5' phosphorylated primer.
