# Supplementary material for: cAMP and c-di-GMP synergistically support biofilm maintenance through the direct interaction of their effectors
Source: Nat Commun. 2022 Mar 21;13:1493. doi: 10.1038/s41467-022-29240-5 (PMC8938473; doi:10.1038/s41467-022-29240-5)
Supplement: Supplementary file 2 — Reporting Summary [file 41467_2022_29240_MOESM2_ESM.pdf]

nature portfolio

Corresponding author(s): Weijie Liu

Last updated by author(s): Mar 2, 2022

Reporting Summary

Nature Portfolio wishes to improve the reproducibility of the work that we publish. This form provides structure for consistency and transparency in reporting. For further information on Nature Portfolio policies, see our Editorial Policies and the Editorial Policy Checklist.

Statistics

For all statistical analyses, confirm that the following items are present in the figure legend, table legend, main text, or Methods section.

n/a	Confirmed	
☐	☒	The exact sample size (*n*) for each experimental group/condition, given as a discrete number and unit of measurement
☐	☒	A statement on whether measurements were taken from distinct samples or whether the same sample was measured repeatedly
☐	☒	The statistical test(s) used AND whether they are one- or two-sided<br>*Only common tests should be described solely by name; describe more complex techniques in the Methods section.*
☒	☐	A description of all covariates tested
☐	☒	A description of any assumptions or corrections, such as tests of normality and adjustment for multiple comparisons
☐	☒	A full description of the statistical parameters including central tendency (e.g. means) or other basic estimates (e.g. regression coefficient) AND variation (e.g. standard deviation) or associated estimates of uncertainty (e.g. confidence intervals)
☐	☒	For null hypothesis testing, the test statistic (e.g. *F*, *t*, *r*) with confidence intervals, effect sizes, degrees of freedom and *P* value noted<br>*Give P values as exact values whenever suitable.*
☒	☐	For Bayesian analysis, information on the choice of priors and Markov chain Monte Carlo settings
☒	☐	For hierarchical and complex designs, identification of the appropriate level for tests and full reporting of outcomes
☒	☐	Estimates of effect sizes (e.g. Cohen's *d*, Pearson's *r*), indicating how they were calculated

Our web collection on statistics for biologists contains articles on many of the points above.

Software and code

Policy information about availability of computer code

Data collection	Biofilm measurement data were acquired using Gen5 (BioTek); Microscale thermophoresis (MST) assay results were acquired using MO.Control/MO.Affinity Analysis Software Bundle.
Data analysis	Statistical analyses were performed using GraphPad Prism (version 9.0.0). Statistical significance was determined using two-sided Student's t-test.

For manuscripts utilizing custom algorithms or software that are central to the research but not yet described in published literature, software must be made available to editors and reviewers. We strongly encourage code deposition in a community repository (e.g. GitHub). See the Nature Portfolio guidelines for submitting code & software for further information.

Data

Policy information about availability of data

All manuscripts must include a data availability statement. This statement should provide the following information, where applicable:

- Accession codes, unique identifiers, or web links for publicly available datasets
- A description of any restrictions on data availability
- For clinical datasets or third party data, please ensure that the statement adheres to our policy

All the data supporting this study are available in the main article, Supplementary Information files, Source Data file, or from the corresponding authors upon reasonable requests (Weijie Liu, leonliu2013@126.com). Source Data are provided with this paper.

Field-specific reporting

Please select the one below that is the best fit for your research. If you are not sure, read the appropriate sections before making your selection.

- [x] Life sciences
- [ ] Behavioural & social sciences
- [ ] Ecological, evolutionary & environmental sciences

For a reference copy of the document with all sections, see nature.com/documents/nr-reporting-summary-flat.pdf

Life sciences study design

All studies must disclose on these points even when the disclosure is negative.

Sample size	Sample sizes for cAMP and c-di-GMP measurement, biofilm biomass, western blot, qPCR, Co-IP were determined according to our extensive laboratory experience and previous studies using these assays (Liu, C. et al. Appl. Environ. Microbiol. 83, e00712-00717 (2017); Almblad, H. et al. J. Bacteriol. 197, 2190-2200 (2015)).
Data exclusions	No data was excluded.
Replication	At least three independent experiments were performed for each assay to verify the reproducibility of the findings.
Randomization	Samples were not allocated to experimental groups.
Blinding	Samples were not allocated to experimental groups.

Reporting for specific materials, systems and methods

We require information from authors about some types of materials, experimental systems and methods used in many studies. Here, indicate whether each material, system or method listed is relevant to your study. If you are not sure if a list item applies to your research, read the appropriate section before selecting a response.

Materials & experimental systems

n/a	Involved in the study	
[ ]	[x]	Antibodies
[x]	[ ]	Eukaryotic cell lines
[x]	[ ]	Palaeontology and archaeology
[x]	[ ]	Animals and other organisms
[x]	[ ]	Human research participants
[x]	[ ]	Clinical data
[x]	[ ]	Dual use research of concern

Methods

n/a	Involved in the study	
[x]	[ ]	ChIP-seq
[x]	[ ]	Flow cytometry
[x]	[ ]	MRI-based neuroimaging

Antibodies

Antibodies used	Monoclonal ANTI-FLAG® M2 antibody produced in mouse (Sigma-Aldrich, Cat#F1804; RRID: AB_262044); IgG from mouse serum (Sigma-Aldrich, Cat#I8765; RRID: AB_1163672); Anti GST-tag mouse monoclonal antibody (CWBio Biosciences, Cat#CW0084; RRID: AB_2892808); Anti His-tag mouse monoclonal antibody (CWBio Biosciences, Cat#CW0286; RRID: AB_2736993); Anti HA-tag mouse monoclonal antibody (CWBio Biosciences, Cat#CW0092; RRID: AB_28929809); Goat anti-mouse IgG, HRP conjugated (CWBio Biosciences, Cat#CW0102; RRID: AB_2736997); Goat anti-mouse IgG-Fc secondary antibody (HRP) (Sino Biological, Cat#SSA006; RRID: AB_2892807).
Validation	All the antibodies used in this study were commercial antibodies, and were validated by the manufactures.
